# Safety of Amulet Left Atrial Appendage Occluder and Watchman Device for Left Atrial Appendage Closure in Patients With Atrial Fibrillation

**DOI:** 10.7759/cureus.55531

**Published:** 2024-03-04

**Authors:** Fadi Sawaya, Bernard Abi-Saleh, Abbas Hoteit, Jennifer Jdaidany, Mohamad B Moumneh, Bernard Harbieh, Maurice Khoury, Salim Aramouni, Farah Abdulhai, Marwan Refaat

**Affiliations:** 1 Cardiology, American University of Beirut Medical Center, Beirut, LBN; 2 Heart and Vascular, Inova, Falls Church, USA

**Keywords:** cardiac arrhythmias, atrial fibrillation, left atrial appendage occlusion, safety, watchman, amulet

## Abstract

Background: Left atrial appendage (LAA) closure is an alternative to chronic anticoagulation for stroke prevention in patients with nonvalvular atrial fibrillation. Multiple devices were used for LAA closure, with the Amplatzer Amulet LAA Occluder (Abbott, Chicago, IL, USA) and Watchman device (Boston Scientific, Marlborough, MA, USA) being the most commonly used in clinical practice. In August 2021, the FDA approved the use of the Amplatzer Amulet LAA Occluder. There is still a knowledge gap in the safety profile of the Amplatzer Amulet LAA Occluder device in comparison to the Watchman device.

Objective: The aim of this study was to assess and compare the safety profile peri-procedure and post-procedure between the Amplatzer Amulet LAA Occluder and Watchman devices.

Methods: Patients who underwent LAA closure using Watchman or Amulet devices from July 2015 to August 2020 at the American University of Beirut Medical Center were included in the analysis. Primary endpoints included peri-operative and post-procedural complications (thromboembolic events, bleeding complications, vascular access complications, pericardial effusion/tamponade, device positional complications and in-hospital death).

Results: The study included 37 patients (21 had Watchman devices, 16 had Amplatzer Amulet LAA Occluder devices, and 28 were men, mean age 76.57 ± 9.3 years). Seven patients developed post-procedural iatrogenic atrial septal defects (four in the Watchman group vs three in the Amulet group, p-value=0.982). Three patients developed pericardial effusion (one in the Watchman vs two in the Amulet group, p-value=0.394). Only one patient developed peri-device leak (one in the Watchman group vs none in the Amulet group, p-value=0.283). One device could not be deployed (one in the Amulet group vs none in the Watchman group, p-value=0.191). None of the patients developed in-hospital death, cardiac tamponade, device embolism, device thrombosis, stroke/transient ischemic attack (TIA), cranial bleeding, or arrhythmias after the procedure. The rate of peri-operative complications was similar between both groups. Both groups displayed low rates of adverse events in the peri-operative and post-operative periods.

Conclusion: There was no significant difference in the safety profile of Amplatzer Amulet LAA Occluders and Watchman devices. There was a low incidence of peri-operative and post-operative adverse events with the implanted devices.

## Introduction

Atrial fibrillation (AF) is the most common arrhythmia and a known cause of cardiogenic ischemic stroke, increasing the risk by fivefold compared to the general population [1]. Currently, the treatment of choice for patients with AF with a congestive heart failure-hypertension-age ≥75-diabetes mellitus-stroke-vascular disease-age 65-74-sex (CHA2DS2-VASc) score >2 is oral anticoagulation with warfarin or direct oral anticoagulants (DOACs). However, a significant proportion of the population cannot be maintained on these medications due to certain contraindications, such as high bleeding risk and noncompliance. These patients are more prone to develop strokes and thromboembolic events [2].

Multiple treatment strategies were developed as a solution to this problem. They mainly focused on the left atrial appendage (LAA) as a therapeutic target. Although considered a vestigial structure, LAA is the most common site of thrombus formation due to AF, with studies showing it to be the origin in more than 90% of patients. LAA occlusion (LAAO) plays an important role in the prevention of stroke events and may also have a role in decreasing arrhythmia burden and atrial remodeling [3-5]. It is performed by using both surgical and percutaneous techniques. Randomized control trials (RCTs) have shown LAAO percutaneous procedures to be non-inferior to vitamin K antagonists, with lower nonprocedural bleeding and mortality rates recorded. They are also non-inferior to new oral anticoagulants (NOACs) in preventing nonvalvular AF-related cardiac and bleeding occurrences [6].

The most extensively studied LAAO device is the Watchman nitinol cage percutaneous LAA closure device. It was approved by the FDA in 2015 for patients with nonvalvular AF at risk for stroke without contraindication to anticoagulation [7]. Unfortunately, most of the studies on the Watchman device (Boston Scientific, Marlborough, MA, USA) were from patients eligible for oral anticoagulation and not from those where oral anticoagulation was contraindicated. An important randomized control trial, the PROTECT AF trial, showed that nitinol cage percutaneous LAA closure was non-inferior to warfarin for cardiovascular (CV) mortality, all-cause mortality, and systemic thromboembolism. However, multiple adverse events related to the procedure were reported, such as pericardial effusion, stroke, and major bleeding. Subsequent studies also showed promising results regarding thromboembolic events, mortality, and major bleeding, particularly hemorrhagic stroke. They also showed a decrease in the rate of procedure-related adverse events, which was attributed to operator experience [8,9]. The Amulet, also known as the Amplatzer Cardiac Plug 2 (ACP 2) (Abbott, Chicago, IL, USA), is another percutaneous device used in the occlusion of the LAA. It has improvements compared to its predecessor, the ACP, but retains ACP1’s basic structure [10]. Few studies evaluating the efficacy of this device exist. Such studies include a few case series that showed success rates of 100% and 96% and only one case that showed pericardial effusion [11,12].

Even though the Watchman remains the most thoroughly examined device and the Amulet has been shown to have prominent success rates and minimal complications, data is still limited, and more studies are required to provide further information concerning safety and efficacy for device use in LAA closure. The aim of this study is to evaluate and compare the different outcomes and safety of these procedures. This article was previously presented as a meeting abstract at the 2022 Heart Rhythm Society Annual Scientific Meeting on May 1, 2022.

## Materials and methods

Study design

This is a single-center observational retrospective study comparing the clinical outcomes of patients who underwent LAA closure using Watchman vs Amulet devices. The study was conducted at the American University of Beirut Medical Center (AUBMC), which is a tertiary care hospital serving a large urban population in a developing country, Lebanon. 

Study population

Our study included a total of 37 consecutive patients who underwent LAA closure procedures using Watchman or Amulet devices between July 2015 and August 2020 at the AUBMC. Inclusion criteria were: (1) all ages, (2) both genders, (3) paroxysmal, permanent, or persistent AF, (4) CHA2DS2-VASc ≥ 2, and (5) contraindication to oral anticoagulation. Experienced cardiologists at our center performed the LAA closure, and all procedural choices of equipment, technique, and pharmacotherapy were chosen. All participants signed a written informed consent for the procedure. The study was approved by the Institutional Review Board of the American University of Beirut. 

Data collection and statistical analysis 

All data were collected retrospectively from the patient's medical records and the cardiac catheterization records at our medical center. Retrieved data included patients’ baseline demographics, medical and surgical histories, procedure characteristics, and periprocedural clinical outcomes. The collected information was filled out in an Excel sheet (Microsoft Corporation, Redmond, Washington, United States) in which each subject was assigned a unique I.D. without any breach of patient confidentiality. 

Data management and analyses were carried out using the SPSS-Version 27.0 (Released 2011; IBM Corp., Armonk, New York, United States). Descriptive statistics were carried out by calculating the number and percent for categorical variables and the mean and standard deviation for continuous ones. Pearson’s chi-square test was conducted to compare the categorical clinical outcomes between the two device groups. Two-tailed tests were used, and statistical significance was determined by a p-value <0.05. 

Procedure protocol 

All patients presented with a history of AF and anticoagulation use. Contraindications for anticoagulation were determined by at least two episodes of either major bleeding caused by the medication or an ischemic stroke or embolism despite it. A cooperative heart team consisting of at least two cardiologists reviewed the case of each patient to opt for the LAA closure procedure. The decision whether to use an Amulet or Watchman device was based upon clinician preference, feasibility, and the availability of the device at the time of the procedure. 

## Results

Patient characteristics

From July 2015 to August 2020, 37 patients underwent LAA occlusion at our center. Two types of devices were used: the Amulet Occluder in 43% of patients and the Watchman in 57% of patients. Baseline characteristics and risk factors are summarized in Table 1. There was a significant difference between the two populations in regard to diabetes and anticoagulation. The mean age was 74.2 ± 10 years in the Watchman group and 79.6 ± 6.7 years in the Amulet group. Of the total Watchman population, 76% were male, while 75% were male in the Amulet group. A significantly larger percentage of the Amulet population had diabetes mellitus as a risk factor when compared to those in the Watchman group (63% vs 24%, respectively, p-value=0.017). Conversely, a significantly larger portion of the Watchman group had already been receiving anticoagulation prior to device implantation when compared to those in the Amulet group (86% vs 44%, respectively, p-value=0.006). 

**Table 1:** Demographic data of our study population. AF: atrial fibrillation; BMI: body mass index; CABG: coronary artery bypass graft; CAD: coronary artery disease; CHAD2DS2-VASc: congestive heart failure-hypertension-age ≥75-diabetes mellitus-stroke-vascular disease-age 65-74-sex; N/A: not applicable; PCI: percutaneous coronary intervention; SD: standard deviation.

Characteristics	Watchman, n (%)	Amulet, n (%)	p-value
Age (mean ± SD)	74.2 ± 10.5	79.6 ± 6.7	0.081
BMI (mean ± SD)	27.6 ± 5	27.6 ± 3.8	0.995
Men	16 (76)	12 (75)	0.936
High CHA2DS2-VASc score (>3)	21 (100)	16 (100)	N/A
History of paroxysmal AF	12 (57)	7 (44)	0.615
History of permanent AF	10 (48)	8 (50)	0.890
History of atrial flutter	2 (10)	3 (19)	0.043
History of heart failure	7 (33)	3 (19)	0.336
History of hypertension	18 (86)	14 (88)	0.879
History of CAD	11 (52)	9 (56)	0.821
History of diabetes	5 (24)	10 (63)	0.017
History of PCI	4 (19)	3 (19)	0.982
History of CABG	7 (33)	3 (19)	0.336
On anticoagulation	18 (86)	7 (44)	0.006
On single antiplatelet	5 (24)	2 (13)	0.152
On dual antiplatelet	0 (0)	1 (6.3)	0.258
On beta-blocker	11 (52)	9 (56)	0.737
On anti-arrhythmic	2 (10)	3 (19)	0.729
Indication for device insertion: hemorrhagic	11 (52)	12 (75)	0.591
Indication for device insertion: ischemic	3 (14)	3 (19)	0.729
Indication for device insertion: both hemorrhagic and ischemic	3 (14)	1 (6.3)	0.243

Peri-operative events and complications

Post-procedure complications in the peri-operative period are shown in Table 2. Device implantation was successful in 95% of the participants of the Watchman group and 94% of the participants of the Amulet group. No statistical difference was present between the two devices. Two events of minor bleeding (observed as formation of bruising and minor hematomas around the groin near the access sites), one event of hospital-acquired infection, three events of pericardial effusion (minimal effusion seen on transthoracic echocardiogram (TTE) post-procedure), one event of para-device leak (minimal leak seen on TTE), one event of thromboembolic event after procedure (formation of deep venous thrombosis (DVT) during hospitalization post-procedure), and one event of congestive heart failure occurred in the study population. None of the participants suffered from a transient ischemic stroke, ischemic stroke, device thrombosis, device embolism, valvular damage, major bleeding events, death, or CV death. No significant difference regarding peri-operative complications between the two devices during hospital admission was reported. 

**Table 2:** Periprocedural events during admission. ASD: atrial septal defect; HF: heart failure; N/A: not applicable; TIA: transient ischemic attack.

Characteristics	Watchman, n (%)	Amulet, n (%)	p-value
Success	20 (95)	15 (94)	1
Thromboembolic event after procedure	1 (5)	0 (0)	1
ASD	4 (19)	3 (19)	1
Congestive HF	0 (0)	1 (6)	0.890
Minor bleeding	2 (10)	0 (0)	0.592
Inability to recapture/position device	0 (0)	1 (6)	0.890
Hospital-acquired infection	1 (5)	0 (0)	1
Pericardial effusion	1 (5)	2 (13)	0.805
Para-device leak	1 (5)	0 (0)	1
TIA	0 (0)	0 (0)	N/A
Ischemic stroke	0 (0)	0 (0)	N/A
Device thrombosis	0 (0)	0 (0)	N/A
Device embolism	0 (0)	0 (0)	N/A
Valvular damage	0 (0)	0 (0)	N/A
Major bleeding	0 (0)	0 (0)	N/A
Death	0 (0)	0 (0)	N/A
Cardiovascular death	0 (0)	0 (0)	N/A

Antithrombotic therapy at discharge

Table 3 covers the discharge medications upon which the various patients were given after hospitalization. Most patients (81%) in the Watchman were discharged on anticoagulation, while less than half of the patients (38%) in the Amulet group were discharged on anticoagulation. Similar percentages in both the Amulet and Watchman populations (38% and 33%, respectively) were discharged on single antiplatelet therapy and beta-blockers (75% and 71%, respectively). A larger portion of the Amulet population was discharged on dual antiplatelets (50%) and antiarrhythmic medication (38%) when compared to patients in the Watchman group (19% dual antiplatelet and 14% antiarrhythmic).

**Table 3:** Medication on discharge.

Discharge	Watchman, n (%)	Amulet, n (%)
Anticoagulation	17 (81)	6 (38)
Single antiplatelet	7 (33)	6 (38)
Dual antiplatelet	4 (19)	8 (50)
Beta-blocker	15 (71)	12 (75)
Antiarrhythmic	3 (14)	6 (38)

## Discussion

This single-center retrospective study, analyzing a population of patients with AF who had interventional occlusion of the LAA, showed good effectiveness and safety profiles when using both devices. Similar rates of peri-operative and post-procedural complications were reported with no significant difference. 

Our study showed similar rates of success in device deployment between the Watchmen and Amulet (95% vs 94%, respectively, p-value=1). Similar success rates of device deployment have been published in previous literature [13-16]. In our population, one Watchman device was not deployed due to the risk of perforation, whereas one Amulet device deployment was aborted due to an unstable device position in the LAA. 

In terms of safety, the rate of post-procedural outcomes was similar between both devices. None of the patients in both groups had any major bleeding events. This is in contrast to findings in Fastner et al.’s registry, where rates of 4.6% were reported [15]. Periprocedural major bleeding event rates were also significantly higher in the Amulet group when compared to the Watchman FLX (2.3% vs 0.1%, respectively, p-value=0.01) in the registry, which differs from our cohort [17]. This can be explained by the fact that we used the Watchman FLX in our study, which is a newer version of the Watchmen device. This might have influenced the safety profile and complication rates when comparing both devices. 

To add to that, peri-device leak (PDL) is a significant limitation and complication of LAAO. In our study, none of the patients showed any significant PDL (one patient had minimal PDL) when they were screened by TTE post-procedure. This is slightly similar to some established data. Ledwoch et al. reported a low rate of PDL (<5%) with a 0% rate of significant PDL in both Watchman and Amulet/ACP devices [18], and Fastner et al. showed that PDL was infrequent in both device implantations after a six-month follow-up [15].

However, the fact that both devices had similarly negligible rates of PDL contrasts with some of the current evidence. Mansour et al. reported a higher rate of significant PDL in the Watchman group when compared to the Amulet group. A possible explanation for this contrast is the difference in the study design. While their study was a double-blinded randomized analysis of device implantation using cardiac coronary tomography angiography (CCTA) or a TEE with follow-up imaging occurring at eight weeks after admission [19], our study focused on immediate peri-operative outcomes with imaging screening done through a TTE. In addition, our results differed from those of a meta-analysis. In the aforementioned study, Amulet devices had a higher incidence of PDL >5 mm when compared to Watchman FLX. Such findings are due to the use of Watchman FLX, the newer generation of Watchman devices, that has improved safety, stability, and occlusion [17]. It is important to note that PDL has been observed to decrease in incidence with time on repetitive follow-up and imaging [20].

A point of concern when considering LAAO is possible device thrombosis, which is associated with an increased incidence of strokes and transient ischemic attacks (TIAs) [21]. The rate of device thrombosis in the literature is not rare. Bai et al. reported a 4.49% incidence rate of device thrombosis post-LAAO. The thromboses were detected at multiple time periods after insertion (45 days, 46 days-six months, six months-one year, >1 year) [22]. While our subjects did not show device thrombosis, screening was done only during admission, and there was no follow-up afterward; hence, assessment of device thrombosis was limited. A similar rate of device thrombosis (3%) was also seen in the registry by Radinovic, with no difference between devices. Patients were discharged mostly on single antiplatelet therapy [6]. Antithrombotic therapy on discharge is still debated due to the risk of bleeding. While a large portion of our subjects were discharged on anticoagulation, Simard et al. reported that discharge medications did not impact device thrombosis [23]. In fact, the Amulet IDE trial showed that patients with Amulet Occluder who were discharged on anticoagulation had higher rates of late pericardial effusion when compared to those who were only given antiplatelet therapy [9].

Limitations

This is a single-center, retrospective observational study that lacked a control group, which makes it difficult to compare the outcomes and assess the effectiveness of the treatment. The small sample size and limited number of patients recruited from a single tertiary center decrease the generalizability of our results. Patients were not followed up in a prospective manner, which impacted the scope of the paper. The baseline populations were not identical, where a significantly larger portion of the Amulet group had diabetes as a risk factor and a larger portion of the Watchman group had already been receiving anticoagulation prior to device insertion. Such factors might affect the outcome of our results.

## Conclusions

While the current retrospective single-center study comparing Amulet with Watchman devices showed comparatively similar periprocedural outcomes and peri-operative complications between both devices, certain limitations exist. These include the lack of long-term follow-up data that demonstrates the durability of the treatment response and the potential for recurrence, as well as the potential for confounding factors or alternative explanations for the patient's symptoms, which could impact the validity of the diagnosis and treatment approach. As a result, additional studies are needed to support the utilization of Amulet devices for LAAO and the notion of its FDA approval.
